# *Escherichia coli* community‐acquired necrotizing pneumonia, an uncommon presentation of a common pathogen: A case report and literature review

**DOI:** 10.1002/rcr2.70015

**Published:** 2024-09-02

**Authors:** Alireza Mohammad Hosseini, Parisa Farshchi, Hanieh Hosseini, Fatemeh Zarei

**Affiliations:** ^1^ Department of Internal Medicine Tehran University of Medical Sciences (TUMS), School of Medicine, Imam Khomeini Hospital Complex Tehran Iran

**Keywords:** community‐acquired pneumonia, *Escherichia coli*, hemoptysis, necrotizing pneumonia

## Abstract

Community‐acquired necrotizing pneumonia is a rare but potentially fatal infection, mainly caused by specific pathogens such as *Streptococcus pneumoniae*, *Staphylococcus aureus*, *Klebsiella pneumoniae*, *Haemophilus influenzae*, and *Pseudomonas aeruginosa*. *Escherichia coli* is extremely rare as a pathogen for community‐acquired necrotizing pneumonia, typically accompanied with bloodstream infection. Here, we report an unusual case of a 60‐year‐old man with uncontrolled diabetes mellitus and no bloodstream infections, who had severe necrotizing *E. coli* pneumonia leading to massive hemoptysis and death. Clinicians should be aware of this pathogen in respiratory infections, as it requires immediate pathogen detection and usually aggressive antibiotic treatment.

## Graphical abstract

Here, we report an unusual case of a 60‐year‐old man with uncontrolled diabetes mellitus and no bloodstream infections, who had severe necrotizing *Escherichia coli* pneumonia leading to massive hemoptysis and death. Clinicians should be aware of this pathogen in respiratory infections, as it requires immediate pathogen detection and usually aggressive antibiotic treatment.

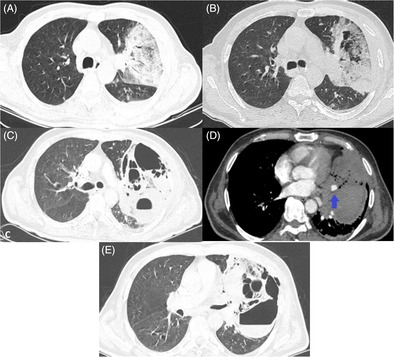

## INTRODUCTION

Community‐acquired pneumonia (CAP) is a worldwide leading cause of mortality and morbidity with a high clinical burden.^1^ Necrotizing pneumonia (NP), though rare, is a severe and highly fatal complication of CAP in which bacterial toxins, inflammatory responses and impaired pulmonary vasculature result in lung parenchymal infarction, necrosis, and cavity formation.^2^ The most common pathogens responsible for NP are *Streptococcus pneumoniae* and *Staphylococcus aureus* followed by *Klebsiella pneumoniae*, *Haemophilus influenzae*, and *Pseudomonas aeruginosa*.^3^ Here, we report an extremely rare and unusual case of community‐acquired necrotizing pneumonia (CANP) induced by *Escherichia coli* with sterile blood culture.

## CASE REPORT

**FIGURE 1:**
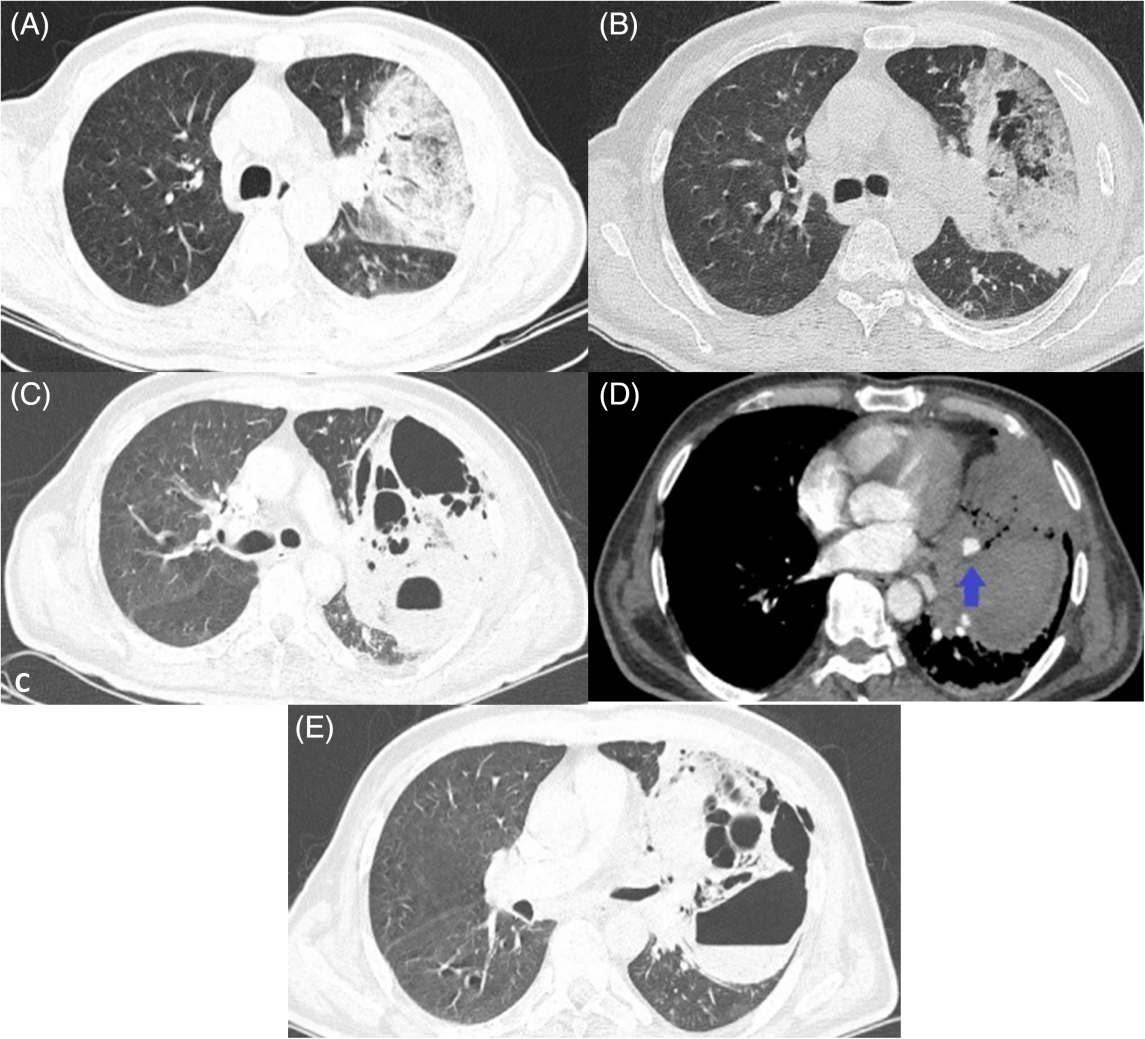
Serial pulmonary imaging during the admission time. (A) Airspace nodular opacity with air bronchogram in left lung upper lobe. (B) Early phase of cavitary formation. (C) Several cavitary foci formation. (D) Pulmonary lingual artery necrosis with perivascular hematoma (arrow). (E) Cavity and air foci expansion.

A 60‐year‐old man was admitted to hospital with complaints of progressive dyspnea, productive cough, and left‐sided pleuritic chest pain from 2 weeks prior to hospitalization. Past medical history was notable for uncontrolled Diabetes Mellitus (DM) and hypertension with no adherence to any medication. He was smoker and opium addicted but did not mention alcohol or other illicit drug abuse, and had no history or clinical manifestations of Chronic Obstructive Pulmonary Disease (COPD). There were no recent similar symptoms in his family members, co‐workers, and his passengers at work. On initial evaluation, the patient was fully conscious and alert, but febrile with a body temperature of 38°C. His blood pressure was 160/90 mmHg, pulse rate 95 beats/min, respiratory rate 20 breaths/min and oxygen saturation 90% while breathing ambient air. Respiratory examination revealed coarse crackles in the left hemithorax and generalized end‐expiratory wheezing in both lungs. The initial laboratory results were remarkable for severe leukocytosis (white blood cell count of 27.1 × 10^3^/μL) and noticeably increased C‐reactive protein (91 mg/L) and erythrocyte sedimentation rate (86 mm/h). Chest computed tomography (CT) scan demonstrated confluent airspace nodular opacity in the left lung upper lobe (LUL), suggesting pneumonia (Figure 1A). Upon diagnosis of CAP, samples for sputum and blood culture were obtained, and immediately thereafter empiric antibiotics were administered using Intravenous (IV) Ampicillin‐sulbactam 3 g every 6 h and oral Azithromycin 500 mg every 24 h.

Despite broad‐spectrum antibiotic therapy for about 72 h, the patient's condition deteriorated. Follow‐up chest CT scan showed worsened lung consolidation with areas of cavitation indicating NP, along with a mass‐like lesion in the left hilum (Figure 1B). Moreover, sputum smear became positive for gram‐negative bacilli. Considering Pseudomonas aeruginosa as an important potential cause, the antibiotic regimen was switched immediately to IV Meropenem 1 g every 8 h and IV Levofloxacin 750 mg every 24 h, while waiting for sputum culture results. IV Vancomycin 20 mg/kg every 12 h was also added due to NP and possible Methicillin‐resistant Staphylococcus aureus colonization, and the patient was scheduled for bronchoscopy. Bronchoalveolar lavage (BAL), endobronchial biopsy, and mediastinal lymph node trans‐bronchial biopsy via endobronchial ultrasonography (EBUS‐TBNA), revealed negative results for malignant cells or granuloma formation. However, all the lavage and biopsy samples showed evidence of acute inflammation, with an unexpected positive culture for *E. coli*, and no other findings suggestive of any fungi, Mycobacterium Tuberculosis, Nocardia, other Mycobacteria, and atypical microorganisms' colonization. The sputum culture also returned positive for *E. coli*, though the blood cultures were all negative. Patient's antibiotics were then adjusted to IV Meropenem 1 g every 8 h and IV Amikacin 20 mg/kg every 24 h based on BAL and sputum culture and antibiotic susceptibility test result.

Further laboratory results showed a significantly high HbA1C (14.3%) indicating severely uncontrolled DM, after which insulin therapy was initiated. Other tests including nasopharyngeal PCR for SARS‐COV‐2, influenza A, and influenza B as well as serum HIV Ag/Ab, HBS Ag, and anti‐HCV Ab were all negative.

Two days after bronchoscopy, patient's condition became severely complicated with an abrupt hemoptysis of about 200 mL. Pulmonary and bronchial CT angiogram demonstrated ground‐glass opacities with several cavitary foci in LUL, with multiple centrilobular nodules indicating trans‐bronchial infection spread. Remarkable evidence of pulmonary lingula artery necrosis was seen, along with hematoma in vicinity of the artery (Figure 1C,D). Interventional radiology was consulted and the artery was ablated, thereafter.

During the 5 days after ablation, non‐massive brown‐coloured hemoptysis continued to appear each day. Follow‐up CT angiogram showed cavity expansion as well as air foci in the chest wall which was indicative of extra‐thoracic expansion of the infection, and no new arterial extravasation were observed (Figure 1E). Despite antibiotic therapy as well as supportive treatment to subside hemoptysis, the patient had a cardiac arrest following a massive episode of hemoptysis (around 600 mL), and died.

## DISCUSSION

*E. coli* is a gram‐negative facultative anaerobe bacillus which belongs to the Enterobacteriaceae family. It is a common non‐pathogenic resident of humans' gastrointestinal tract and can be transmitted via faecal‐oral transmission.^4^ However, pathogenic *E. coli* strains often lead to urinary and gastrointestinal (GI) tract infections as well as septicemia in both healthy and immunocompromised population,^5^ and some recent cases of ventilator‐associated pneumonia (VAP).^6^ A recent genetic analysis identified Pneumonia‐causing pathogenic *E. coli* cases with their main characteristics defining them as extra‐intestinal.^7^ Despite *E. coli*'s prevalence in the aforementioned infections, it is found to be quite rare in CAP and even more so in NP.^8^, ^9^

Only a few studies have reported *E. coli* as responsible for CANP. Khalafi et al. reported a case of severe NP due to *E. coli* in a 44‐year‐old man with uncontrolled DM (HbA1C of 17.6%). The patient presented with diarrhoea and urinary urgency in addition to respiratory symptom, as well as positive blood culture for *E. coli*, illustrating simultaneous bacteremia and possible *E. coli*‐induced urinary tract and GI infection. The patient went on mechanical ventilation but fortunately was weaned off and discharged from hospital.^10^ Harsha et al. described a 67‐year‐old man with uncontrolled DM complaining from a one‐month history of fever, cough, and lower back pain. Investigations revealed bilateral NP in chest CT scan and *E. coli* in BAL, though blood cultures were all negative. The patient improved clinically and within 4 months NP cavities were resolved.^11^ Jaffey et al. reported a case of fulminant lobar pneumonia in a 37‐year‐old intellectually disabled, immunocompetent man who died only 2 days after symptoms initiated. Antemortem and post‐mortem blood samples as well as lung specimens showed positive cultures for *E. coli*.^12^ Ganipisetti et al. described a 62‐year‐old woman with history of DM, asthma, and hypertension presenting with chills, cough, and malaise from 5 days prior to admission. Imaging showed bibasilar infiltrates and blood cultures became positive for *E. coli* with simultaneous intestinal inflammation. The patient was discharged with stable condition and low‐flow oxygen demand.^13^

In this article we report an extremely rare, aggressive, and fatal case of *E. coli* CANP. Despite timely diagnosis and appropriate treatment, the patient expired due to fulminant and unexpected course of the disease. A uniqueness of the present case was that the patient did not have any simultaneous urinary or GI infection with *E. coli*, nor was blood culture positive for *E. coli*. This is in stark contrast with reviewed literature, as they all showed either *E. coli* bacteremia and/or extra‐pulmonary *E. coli* infections. Another point of significance was that the patient was poor‐controlled diabetic similar to most cases available in the literature.

In diagnosis of CAP, in particular CANP, we should consider *E. coli* as an aggressive and even fatal pathogen, in order to reach timely diagnosis and initiate appropriate treatment to reduce morbidity and mortality. We should also consider DM, in particular uncontrolled DM, as a significant risk factor for *E. coli* CANP. It is important to note that *E. coli*‐induced NP does not necessarily accompany with bacteremia and/or extrapulmonary infections, thus lack of septicemia should not mislead us to rule out *E. coli* as the root cause of CANP.

*E. coli* CANP is an extremely rare infection, but is potentially aggressive and fatal, mainly prevalent in poor‐controlled diabetic patients. Clinicians should be aware of this pathogen in respiratory infections, as it requires immediate pathogen detection followed by appropriate antibiotic treatment. Future studies can reveal useful epidemiologic, diagnostic, and therapeutic aspects of *E. coli CANP*.

## AUTHOR CONTRIBUTIONS

Parisa Farshchi and Alireza Mohammad Hosseini were responsible for conceptualization and patient data collection, and wrote the main manuscript. Fatemeh Zarei conducted literature review and was involved in manuscript writing. Hanieh Hosseini reviewed the final manuscript. All the authors critically reviewed, edited and approved the final manuscript.

## CONFLICT OF INTEREST STATEMENT

None declared.

## ETHICS STATEMENT

The authors declare that appropriate written informed consent was obtained for the publication of this manuscript and accompanying images.

## Data Availability

Data available on request from the authors.
